# Hydroxysafflor Yellow A Ameliorates Myocardial Ischemia/Reperfusion Injury by Suppressing Calcium Overload and Apoptosis

**DOI:** 10.1155/2021/6643615

**Published:** 2021-05-21

**Authors:** Jingxue Ye, Ruiying Wang, Min Wang, Jianhua Fu, Qiong Zhang, Guibo Sun, Xiaobo Sun

**Affiliations:** ^1^Institute of Medicinal Plant Development, Chinese Academy of Medical Sciences & Peking Union Medical College, Beijing 100193, China; ^2^Beijing Key Laboratory of Innovative Drug Discovery of Traditional Chinese Medicine (Natural Medicine) and Translational Medicine, Beijing 100193, China; ^3^NMPA Key Laboratory for Research and Evaluation of Pharmacovigilance, Beijing 100193, China; ^4^Xiyuan Hospital, China Academy of Chinese Medical Sciences, Beijing 100091, China

## Abstract

Myocardial ischemia/reperfusion injury (MI/RI) is an urgent problem with a great impact on health globally. However, its pathological mechanisms have not been fully elucidated. Hydroxysafflor yellow A (HSYA) has a protective effect against MI/RI. This study is aimed at further clarifying the relationship between HSYA cardioprotection and calcium overload as well as the underlying mechanisms. We verified the protective effect of HSYA on neonatal rat primary cardiomyocytes (NPCMs) and human-induced pluripotent stem cell-derived cardiomyocytes (hiPSC-CMs) from hypoxia-reoxygenation (HR) injury. To explore the cardioprotective mechanism of HSYA, we employed calcium fluorescence, TUNEL assay, JC-1 staining, and western blotting. Finally, cardio-ECR and patch-clamp experiments were used to explain the regulation of L-type calcium channels (LTCC) in cardioprotection mediated by HSYA. The results showed that HSYA reduced the levels of myocardial enzymes and protected NPCMs from HR injury. HSYA also restored the contractile function of hiPSC-CMs and field potential signal abnormalities caused by HR and exerted a protective effect on cardiac function. Further, we demonstrated that HSYA protects cardiomyocytes from HR injury by decreasing mitochondrial membrane potential and inhibiting apoptosis and calcium overload. Patch-clamp results revealed that MI/RI caused a sharp increase in calcium currents, which was inhibited by pretreatment with HSYA. Furthermore, we found that HSYA restored contraction amplitude, beat rate, and field potential duration of hiPSC-CMs, which were disrupted by the LTCC agonist Bay-K8644. Patch-clamp experiments also showed that HSYA inhibits Bay-K8644-induced calcium current, with an effect similar to that of the LTCC inhibitor nisoldipine. Therefore, our data suggest that HSYA targets LTCC to inhibit calcium overload and apoptosis of cardiomyocytes, thereby exerting a cardioprotective effect and reducing MI/RI injury.

## 1. Introduction

Ischemic heart disease is associated with high mortality rates and disability burden worldwide [1]. Recovery of blood circulation in the ischemic heart through therapy can cause further damage to the myocardial tissue, which is defined as myocardial ischemia/reperfusion injury (MI/RI) [2]. The mechanisms underlying MI/RI are complex and have not yet been fully elucidated. Mechanistically, MI/RI mainly involves oxidative stress, calcium overload, inflammation, and energy metabolism disorders [3]. Oxidative stress causes cell membrane damage, which in turn triggers calcium overload. In addition, mitochondrial dysfunction can induce calcium overload in the mitochondria, thereby disrupting calcium homeostasis in the cell [4, 5]. Therefore, calcium overload is considered as an intermediate link in the MI/RI mechanism. The intracellular [Ca^2+^]_i_ affects the excitation-contraction coupling of cardiomyocytes. Calcium channel proteins such as SERCA, RyR2, NCX, and L-type calcium channels (LTCC) work together to maintain the intracellular calcium homeostasis [6]. In addition, mitochondria are also important places for Ca^2+^ storage and transportation, and Ca^2+^ in the mitochondria is also involved in the process of oxidative phosphorylation and ATP formation in mitochondria [7]. Importantly, LTCC are important to transport extracellular Ca^2+^ into cardiomyocytes, and disorders of their structure or function can also cause an imbalance in intracellular calcium homeostasis [8]. In addition, LTCC inhibitors, such as diltiazem, have been shown to greatly improve MI/RI [9]. Thus, identifying candidates that can inhibit LTCC may be a reasonable and promising strategy to treat MI/RI.

Hydroxysafflor yellow A (HSYA) is the dominant water-soluble component of *C. tinctorius* L., which is used for treating cardiovascular diseases [10]. Notably, HSYA injection has been clinically used in China to treat stable fatigue angina caused by blood stasis [11]. Our team proved that HSYA plays a protective role against MI/RI and cardiomyocyte hypoxia-reoxygenation (HR) injury [12, 13]. Proven protective mechanisms mainly include inhibition of apoptosis, inflammation, mitochondrial dysfunction, and activation of autophagy [14, 15]. For example, HSYA protects against HR injury via Akt/hexokinase II, independently of the ERK/GSK-3*β* pathway [16], and also displays a cardioprotective effect in vitro and in vivo via suppression of the JAK2/STAT1 pathway [15]. Moreover, HSYA treatment inhibits LTCC and promotes vasodilation in hypertensive rats [17]. HSYA can also improve diabetic cardiac insufficiency by regulating calcium homeostasis in rats [18]. However, whether HSYA can reduce MI/RI by regulating LTCC is currently unknown.

Our research team has previously shown that HSYA protects against MI/RI in rats and HR in H9C_2_ cardiomyocytes. In this study, we used neonatal rat primary cardiomyocytes (NPCMs) and human-induced pluripotent stem cell-derived cardiomyocytes (hiPSC-CMs) to further demonstrate that HSYA exerts a protective effect against MI/RI by regulating LTCC.

## 2. Materials and Methods

### 2.1. Materials and Experimental Animals

HSYA was purchased from Chengdu Mansite Biotechnology Co., Ltd. (Chengdu, China). Sprague Dawley (SD) rats were purchased from SPF (Beijing) Biotechnology Co., Ltd. (Beijing, China). Rats were housed in 60% humidity and at approximately 25°C with a 12 h light-dark period and were fed to meet the requirements of animal welfare. All procedures complied with the Guide for the Care and Use of Laboratory Animals. All experiments were approved by the Laboratory Animal Ethics Committee of the Institute of Medicinal Plant Development, Chinese Academy of Medical Sciences.

### 2.2. Culture and Treatment of NPCMs

Refer to a previous study for specific steps of NPCM culture using enzymatic hydrolysis [19]. The hearts of one-day-old rats were removed and digested with 1% type II collagenase and trypsin without EDTA (2 : 1). After 1.5 h of natural sedimentation, the myocardial fibrocytes were discarded to obtain ventricular myocytes. The cells began to beat synchronously for 1-2 days after plating, and the experiment was initiated.

The cells were grouped as follows: control group, HR group, HR+HSYA (2.5, 5, and 10 *μ*M) group, and HR+nisoldipine. To establish the HR model, after replacing the medium with DMEM without glucose, NPCMs were placed in an anaerobic glove box (Type C, Coy Laboratory, CA, USA) for 6 h. To stimulate reoxygenation, NPCMs were provided with fresh DMEM and placed in a 37°C incubator. Cells were pretreated with HSYA (2.5, 5, and 10 *μ*M) and nisoldipine (100 nM) for 4 h before establishing HR [12, 13].

### 2.3. Cell Viability Assay

Cell viability was detected by the Cell Counting Kit-8 (CCK8) method as previously reported [12]. NPCMs were seeded into 96-well plates. After various interventions, CCK8 (10 *μ*L/well) was added to the cells and then reacted with cells for about 2 h at 37°C. And the cells were measured at 570 nM using a Beckman AU480 microplate reader (Fullerton, CA, USA).

### 2.4. Indicators of NPCM Myocardial Injury

NPCMs were seeded into 6-well plates. After various treatments, the degree of myocardial damage in NPCMs was assessed using the aspartate aminotransferase (AST) kit, lactate dehydrogenase (LDH) kit, and creatine kinase-MB (CK-MB) kit (Nanjing Jiancheng Bioengineering Institute, Nanjing, China). The experiment was carried out according to the manufacturers' instructions.

### 2.5. Simultaneous Detection of hiPSC-CM Contraction and Field Potential Indicators

hiPSC-CMs were purchased from NEXEL Cardiosight-S (Cat# C-002). The e-plate was covered with fibronectin (50 *μ*L/mL) in 37°C for 2 h. The hiPSC-CMs were thawed quickly in a 37°C water bath and seeded at 1 × 10^5^ cells/well in culture plates. The medium was replaced with fresh medium the next day and thereafter changed every two days. On the e-plates, beat signals and potential signals of hiPSC-CMs were monitored in real time using the xCELLigence RTCA Cardio ECR System. Drug treatments started after the beat signals, and potential signals became stable. The characteristics of the drug were reflected by changes in the beat signals and potential signals [20].

### 2.6. MI/RI Model and Rat Ventricular Myocyte Isolation

MI/RI model establishment and rat ventricular myocyte isolation were performed as previously reported [21]. SD rats (270–280 g) were anesthetized using sodium pentobarbital (50 mg/kg). The rats were connected to a ventilator after tracheal intubation. A 6-0 silk thread was passed into a needle which was subsequently passed under the left coronary artery at a depth of 1-1.5 mm for a distance of 3-4 mm. After removal of the needle, leaving the thread under the artery, both ends of the thread were passed through a 3.5 cm long disposable intravenous infusion tube. For ischemia, the tube was gently pushed along the thread to tighten the thread around the coronary artery. The thread was clamped in place in the tube with a hemostat and left for 30 min of ischemia after which the tube and thread were removed for reperfusion for 24 h. HSYA and HSYA+IR groups were administered HSYA (16 mg/kg) by intravenous tail injection 30 min prior to establishing I/R [13, 15]. The nisoldipine+IR group was administered nisoldipine (2 mg/kg) for three days by gavage prior to I/R. In the sham and HSYA groups, the vessels were not ligated, while the other procedures were the same as those in the IR-receiving groups. Following MI/RI, rat hearts were removed, and single cardiomyocytes were isolated by enzymatic hydrolysis as previously reported [22], which was used in the subsequent patch-clamp experiment.

### 2.7. Patch-Clamp Techniques

Calcium currents were recorded using whole-cell voltage-clamp techniques, as previously reported [23, 24]. Currents were digitized at a sampling rate of 5 kHz. I_Ca, L_ was recorded by 10 mV step depolarizations to different potentials between −60 and +80 mV from a holding potential of −60 mV. The voltage dependence of steady-state inactivation was obtained by a 250 ms test pulse to 10 mV after a prepulse of 2000 ms from −60 mV to +80 mV in 10 mV steps. Recovery from inactivation was analyzed using the double-pulse protocol. From a holding potential of −60 mV, the membrane was first pulsed to 10 mV for 250 s to induce inactivation. The membrane was then repolarized to −80 mV for varying periods before the second pulse to 10 mV for 250 ms was applied.

### 2.8. Intracellular Calcium Detection

Fluo-3 AM (Thermo Fisher, F1242), a calcium fluorescence indicator, was used to measure the intracellular calcium levels of hiPSC-CMs [25, 26]. Following the different group treatments, hiPSC-CMs were incubated in a medium containing Fluo-3 AM (5 *μ*M) in the dark for at least 30 min. After 3 washes with PBS, hiPSC-CMs were placed in a fresh medium for 20 min to ensure the generation of calcium fluorescence. To evaluate intracellular calcium levels, images were captured using an IncuCyte™ S3 ZOOM cell imaging system (Essen BioScience, Ann Arbor, MI).

### 2.9. Terminal Deoxynucleotidyl Transferase dUTP Nick-End Labeling (TUNEL) Assay

Apoptosis of NPCMs was determined using TUNEL assay as previously reported [27, 28]. Following treatments, 0.1% Triton X-100 was used to increase membrane permeability and TUNEL reaction solution was added to the cells for 1 h at 37°C. After washing three times with PBS, images were taken using an IncuCyte™ S3 ZOOM cell imaging system (Essen BioScience, Ann Arbor, MI).

### 2.10. Mitochondrial Membrane Potential Detection

The mitochondrial membrane potential in NPCMs was detected using the JC-1 dye (Thermo Fisher, 65-0851-38) [29]. JC-1 (1 : 1000) was added to NPCMs after treatments and incubated for 30 min at 37°C. Then, cells were washed three times with PBS. Images were collected using an IncuCyte™ S3 ZOOM cell imaging system (Essen BioScience, Ann Arbor, MI).

### 2.11. Western Blotting

Western blot analysis was performed as previously reported [30]. Briefly, 50 *μ*g of total proteins in each lane was separated using 10% SDS-PAGE and transferred to nitrocellulose (NC) membranes. The membrane was blocked in 5% skim milk at room temperature for at least 2 h and then incubated in primary antibody overnight at 4°C. After washing three times with TBS-Tween (TBST), the NC membrane was incubated with the corresponding secondary antibody at room temperature for 2 h. Finally, the bands were visualized using an ECL kit. The primary antibodies used in this study included *α*1C (ab84814, 1 : 1000), tubulin (ab18207, 1 : 2000), *α*2*δ* (A10315, 1 : 500), Bcl-2 (A0208, 1 : 500), Bax (A19684, 1 : 500), caspase-3 (A2156, 1 : 500), and caspase-9 (A2636, 1 : 500).

### 2.12. Data and Statistical Analysis

Data were obtained and analyzed blindly and are presented as the mean ± standard deviation (SD). In the column diagrams, one-way ANOVA followed by Tukey's test was used for multiple comparisons using GraphPad Prism 5.0. In the linear graphs' data, two-way ANOVA was used for multiple comparisons using GraphPad Prism 5.0. Statistical significance was set at *P* < 0.05 (two-tailed).

## 3. Results

### 3.1. HSYA Alleviates HR-Induced Myocardial Injury in NPCMs

**Figure 1:**
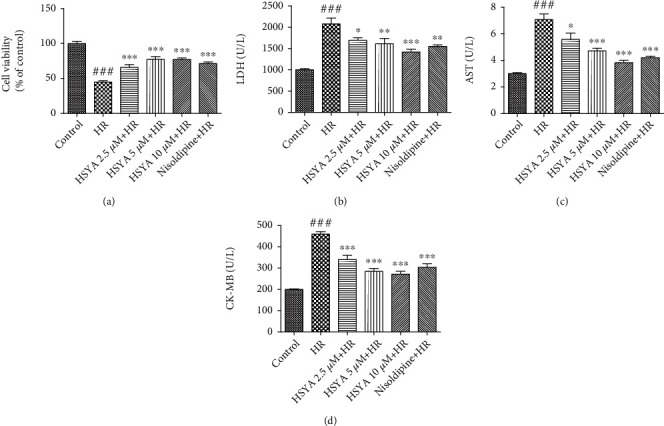
Effect of HSYA on HR-induced myocardial injury in NPCMs. NPCMs were preincubated with HSYA (2.5, 5, and 10 *μ*M) and nisoldipine (100 nM) for 24 h, followed by hypoxia (6 h) and reoxygenation (24 h). (a) Cell viability was detected using the CCK-8 assay. (b–d) Myocardial injury was assessed by detecting lactic dehydrogenase (LDH), aspartate transaminase (AST), and creatine kinase-MB (CK-MB) levels in NPCMs. Data are expressed as the mean ± SD. ^###^*P* < 0.001 vs. control group; ^∗^*P* < 0.05 vs. HR group, ^∗∗^*P* < 0.01 vs. HR group, and ^∗∗∗^*P* < 0.001 vs. HR group.

First, we sought to confirm the cardioprotective effects of HSYA. The effects of HSYA against HR-induced myocardial injury were determined by CCK-8 assay and myocardial enzyme levels. CCK-8 indicated that HSYA (2.5, 5, and 10 *μ*M) dose-dependently attenuated myocardial damage caused by HR (Figure 1(a)). Myocardial cell damage can also cause leakage of myocardial enzymes, including LDH, AST, and CK-MB. We found that HR in NPCMs led to an increase in LDH, AST, and CK-MB levels, which was attenuated by pretreatment with HSYA (2.5, 5, and 10 *μ*M) in a dose-dependent manner (Figures 1(b)–1(d)), consistent with the results of the CCK8 assay. Therefore, these data showed that HSYA significantly alleviated myocardial injury caused by HR.

### 3.2. HSYA Attenuates HR-Induced Myocardial Apoptosis in NPCMs

**Figure 2:**
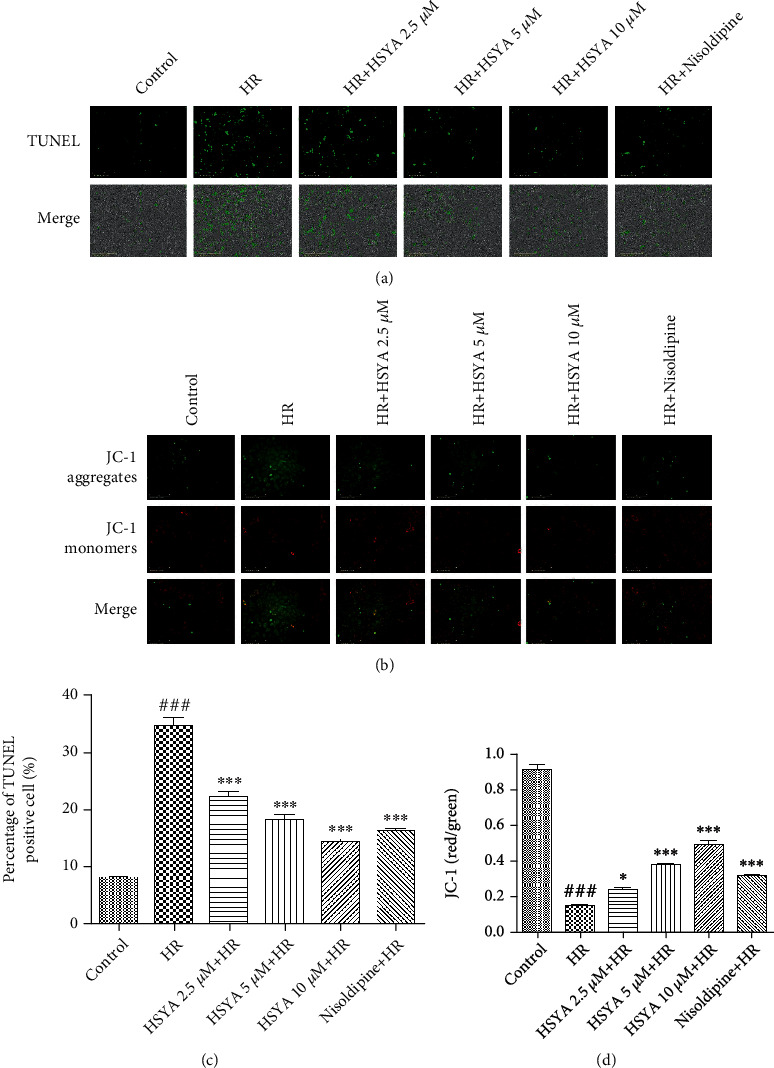
Effect of HSYA on HR-induced myocardial apoptosis in NPCMs. NPCMs were preincubated with HSYA (2.5, 5, and 10 *μ*M) and nisoldipine (100 nM) for 24 h, followed by hypoxia (6 h) and reoxygenation (24 h). (a) Representative images of TUNEL staining (scale bar, 200 *μ*m). (b) Representative images of JC-1 staining (scale bar, 200 *μ*m). (c) Analysis of TUNEL staining results. (d) Analysis of JC-1 staining results. Data are expressed as the mean ± SD. ^###^*P* < 0.001 vs. control group; ^∗^*P* < 0.05 vs. HR group; ^∗∗∗^*P* < 0.001 vs. HR group.

**Figure 3:**
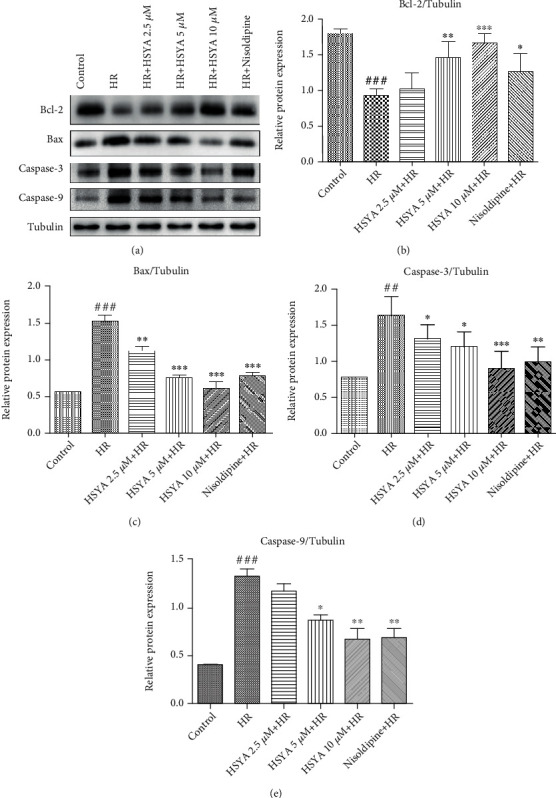
Effect of HSYA on apoptosis protein expressions in heart tissue of NPCMs. NPCMs were preincubated with HSYA (2.5, 5, and 10 *μ*M) and nisoldipine (100 nM) for 24 h, followed by hypoxia (6 h) and reoxygenation (24 h). (a) Representative immunoblots and (b–e) quantification analysis of Bcl-2, Bax, caspase-3, and caspase-9 were shown. Data are expressed as the mean ± SD. ^##^*P* < 0.01 vs. control group, ^###^*P* < 0.001 vs. control group; ^∗^*P* < 0.05 vs. HR group, ^∗∗^*P* < 0.01 vs. HR group, and ^∗∗∗^*P* < 0.001 vs. HR group.

Apoptosis plays an important role in the HR injury of cardiomyocytes. Therefore, we verified the cardioprotective effect of HSYA using TUNEL staining and JC-1 assay. As expected, a large number of TUNEL-positive cells was observed in HR-treated groups, which was reduced by HSYA (2.5, 5, and 10 *μ*M) in a dose-dependent manner (Figures 2(a) and 2(c)). Consistently, the ratio of red and green fluorescence intensity was also significantly higher in the HSYA-treated groups than in the HR group, indicating that HSYA can reduce the depolarization of the mitochondrial membrane and inhibit apoptosis (Figures 2(b) and 2(d)). The levels of Bcl-2, Bax, and caspase most directly indicate the level of apoptosis. In our experiment, HR of NPCMs led to a lower Bcl-2 level and higher Bax, caspase-3, and caspase-9 levels (Figure 3). However, HSYA treatment dose-dependently regulated apoptosis-related protein expression and attenuated myocardial cell apoptosis (Figure 3). Taken together, these data show that HSYA reduces HR-induced myocardial damage by inhibiting apoptosis.

### 3.3. HSYA Mitigates Sarcomere Contraction and Field Potential Abnormality Induced by HR in hiPSC-CMs

**Figure 4:**
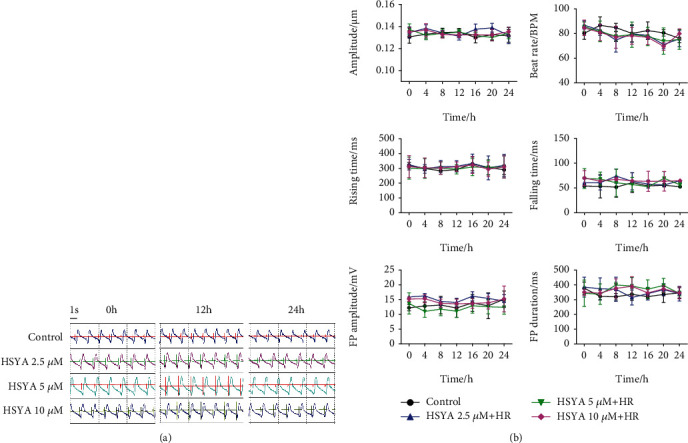
Effect of HSYA on HR-induced abnormal contraction and field potential signals in hiPSC-CMs. Following HSYA (2.5, 5, and 10 *μ*M) treatment for 24 h and hypoxia for 6 h, the impedance and field potential signals of hiPSC-CMs were monitored in real time during reoxygenation. (a) Representative merged images of contraction and field potential signals for 5.4 s. (b) Quantification analysis of contraction amplitude, beat rate, rising time, falling time, FP amplitude, and FP duration. ^#^*P* < 0.05 vs. control group (the color of # is the same as that of the curve); ^∗^*P* < 0.05 vs. HR group (the color of ∗ is the same as that of the curve).

**Figure 5:**
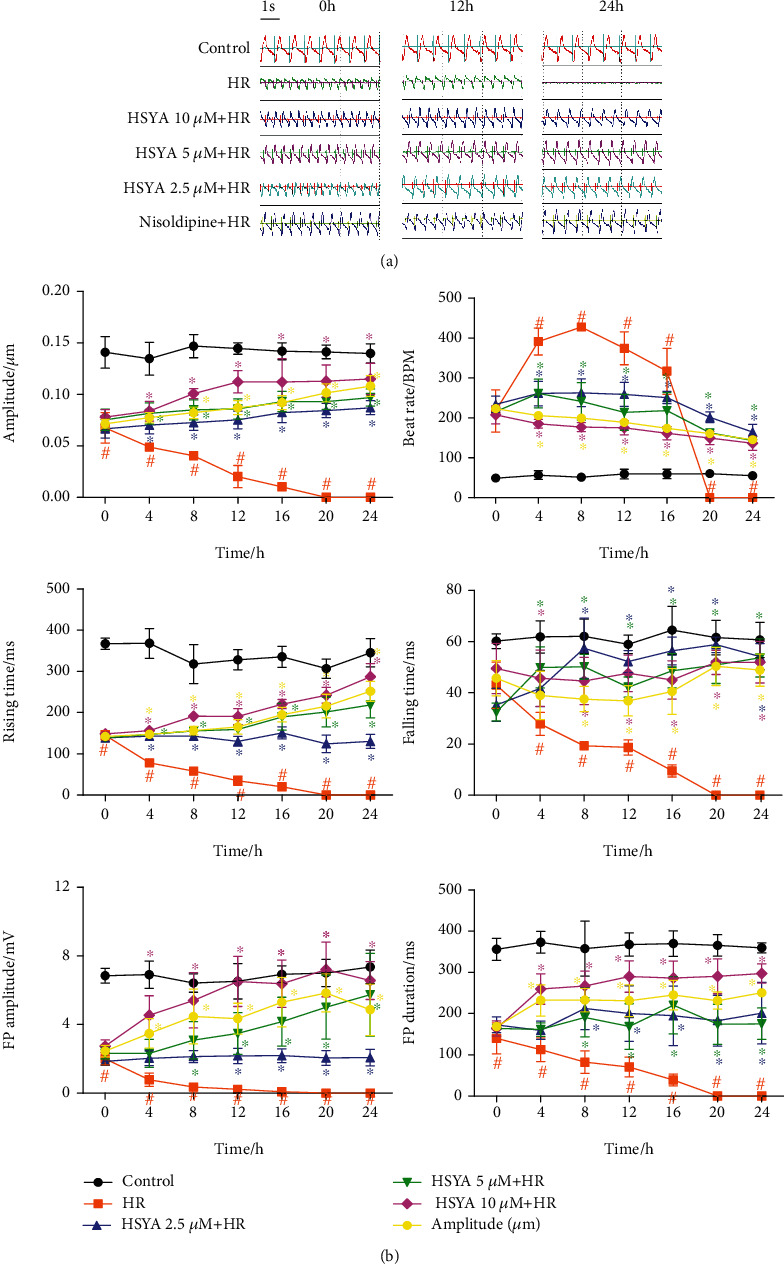
Effect of HSYA on contraction and field potential signals in hiPSC-CMs. The impedance and field potential signals of hiPSC-CMs were monitored in real time after treatment with HSYA (2.5, 5, and 10 *μ*M). (a) Representative merged images of contraction and field potential signals for 5.4 s. (b) Quantification analysis of contraction amplitude, beat rate, rising time, falling time, FP amplitude, and FP duration. ^#^*P* < 0.05 vs. control group (the color of # is the same as that of the curve).

The cardio-ECR system can detect cardiomyocyte contraction and field potential signals in real time. We used this method to further evaluate the effect of HSYA on hiPSC-CMs. Following hypoxia, the electrode plates were immediately placed on the cardio-ECR system for real-time detection in hiPSC-CMs, which continued for 24 h. Within 16 h of reoxygenation, the contraction amplitude of hiPSC-CMs gradually decreased, the beat rate increased, the contraction and relaxation processes became abnormal, and the amplitude and duration of the field potential signal also gradually decreased (Figure 4). After 20 h of reoxygenation, the contraction and field potential indicators of hiPSC-CMs were no longer detected. Subsequently, hiPSC-CMs were treated with HSYA (2.5, 5, and 10 *μ*M) and L-calcium channel inhibitor nisoldipine for 24 h before hypoxia. This process was also continuously monitored on the cardio-ECR system. HSYA treatment did not cause significant fluctuations in contraction and field potential signals in hiPSC-CMs before hypoxia (Figure 5). However, after hypoxia, HSYA treatment significantly enhanced the ability of hiPSC-CMs to resist HR damage. Specifically, HSYA significantly increased the contraction amplitude, reduced the beat rate, restored the normal rhythm of contraction and relaxation, and reversed the field potential signal of hiPSC-CMs (Figure 4). Among the concentrations tested, 10 *μ*M HSYA showed the highest cardioprotective effect. Collectively, the data indicate that HSYA plays a cardioprotective effect by improving contractility and field potential changes in hiPSC-CMs.

### 3.4. HSYA Inhibits HR-Induced Calcium Overload in NPCMs

**Figure 6:**
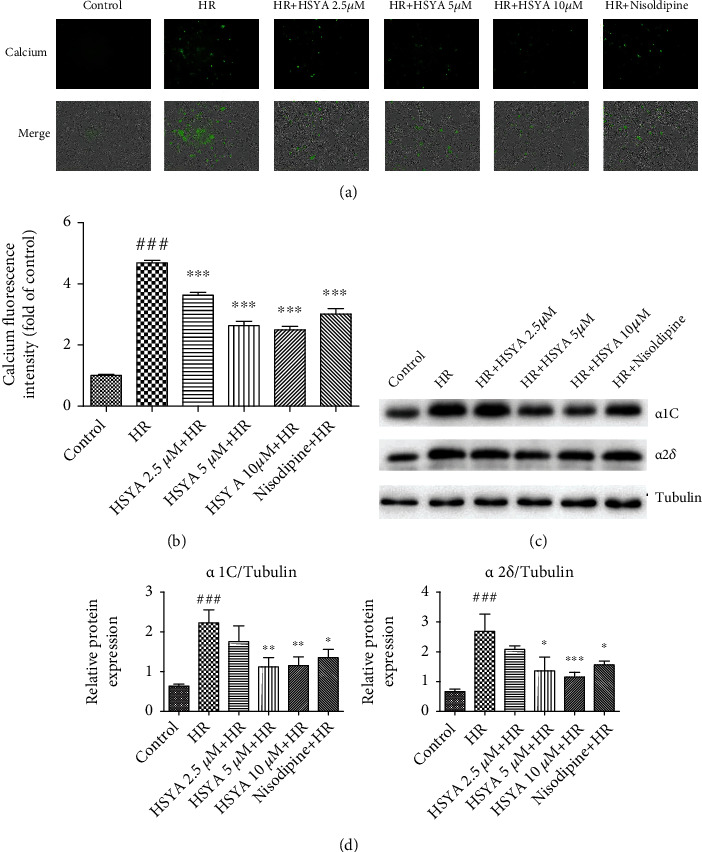
Effect of HSYA on HR-induced calcium overload in NPCMs. NPCMs were preincubated with HSYA (2.5, 5, and 10 *μ*M) and nisoldipine (100 nM) for 24 h, followed by hypoxia (6 h) and reoxygenation (24 h). (a, b) Representative images and analysis results of calcium fluorescence staining (scale bar, 200 *μ*m). (c, d) Representative immunoblots and quantified analysis of *α*1C and *α*2*δ*. Data are expressed as the mean ± SD. ^###^*P* < 0.001 vs. control group; ^∗^*P* < 0.05 vs. HR group, ^∗∗^*P* < 0.01 vs. HR group, and ^∗∗∗^*P* < 0.001 vs. HR group.

Next, we examined the effect of HSYA on HR-induced intracellular calcium overload by analyzing calcium fluorescence. Calcium fluorescence levels were significantly higher in the HR group than in the control group, but the dysregulations were dose-dependently attenuated by HSYA treatment (Figure 6). Several factors cause calcium overload in cardiomyocytes, most prominently LTCCs. We detected the expression of two important subunits of LTCC, *α*1C, and *α*2*δ*. HSYA treatment significantly attenuated the higher expression of LTCC subunits, with an effect comparable to that of LTCC inhibitors. Therefore, our data suggest that HSYA may reduce HR-induced calcium overload by regulating calcium channels.

### 3.5. HSYA Inhibits an Increase of Calcium Current Induced by IR in Rats

**Figure 7:**
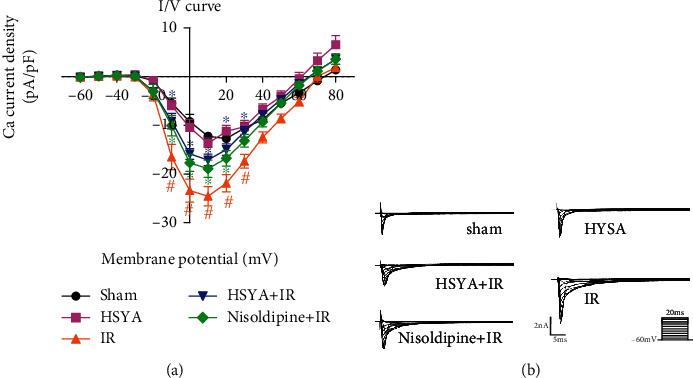
Effect of HSYA on IR-induced elevation of calcium current in rats. After HSYA (16 mg/kg) administration and IR, patch clamp was performed. (a) I–V curve of the Ca^2+^ current was recorded. (b) Representative Ca^2+^ current traces are shown. ^#^*P* < 0.05 vs. sham group (the color of # is the same as that of the curve); ^∗^*P* < 0.05 vs. IR group (the color of ∗ is same as that of the curve).

We then sought to clarify the effect of HSYA on calcium current increase caused by IR. To this aim, we established the MI/RI model in rats by ligating the left anterior descending coronary artery and then isolated a single rat primary cardiomyocyte for patch-clamp experiments. Consistent with the calcium overload caused by HR, the calcium current was higher in the IR group than in the control group (Figure 7). However, pretreatment with HSYA markedly attenuated the increase in calcium current induced by MI/RI. LTCC inhibitors can also reduce calcium currents. This indicates that HSYA may inhibit calcium overload by reducing the opening of LTCC.

### 3.6. HSYA Abolishes Sarcomere Contraction and Field Potential Abnormality Induced by LTCC Agonist in hiPSC-CMs

**Figure 8:**
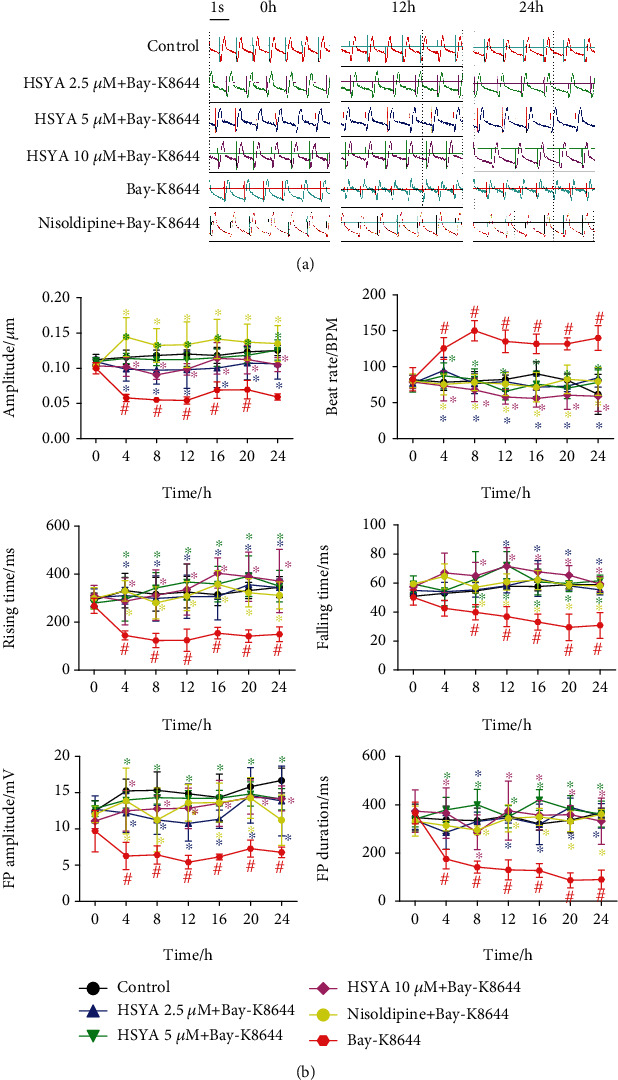
Effect of HSYA on LTCC agonist-induced abnormal contraction and field potential signals in hiPSC-CMs. Following HSYA (2.5, 5, and 10 *μ*M) treatment for 24 h, the impedance and field potential signals of hiPSC-CMs were monitored in real time during Bay-K8644 treatment. (a) Representative merged images of contraction and field potential signals for 5.4 s. (b) Quantification analysis of contraction amplitude, beat rate, rising time, falling time, FP amplitude, and FP duration. ^#^*P* < 0.05 vs. control group (the color of # is the same as that of the curve); ^∗^*P* < 0.05 vs. Bay-K8644 group (the color of ∗ is same as that of the curve).

Bay-K8644 is an LTCC agonist. We then employed the cardio-ECR system to further study the regulation of LTCC by HSYA. Bay-K8644 treatment reduced the contraction amplitude of hiPSC-CMs, raised the beat rate, and lowered the field potential signal. These changes were consistent with those caused by HR. However, HSYA treatment significantly improved contraction and field potential indicators of hiPSC-CMs (Figure 8). This shows that HSYA can protect cardiomyocytes against the electrical signal disturbances caused by the excessive activation of LTCC.

### 3.7. HSYA Reverses the Calcium Current Increase Induced by LTCC Agonist in hiPSC-CMs

**Figure 9:**
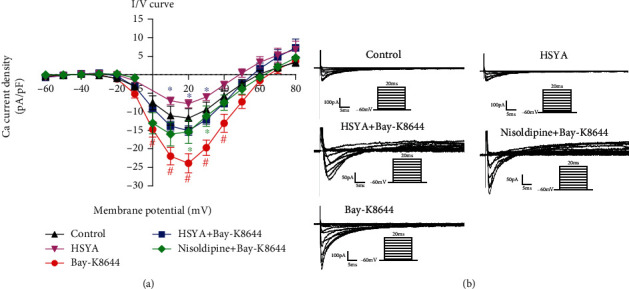
Effect of HSYA on increased calcium current in rats. Patch clamp was performed after HSYA (10 *μ*M) treatment for 24 h and Bay-K8644 (8 nM). (a) I–V curve of the Ca^2+^ current was recorded. (b) Representative Ca^2+^ current traces are shown. ^#^*P* < 0.05 vs. control group (the color of # is the same as that of the curve); ^∗^*P* < 0.05 vs. Bay-K8644 group (the color of ∗ is same as that of the curve).

Finally, we further validated the regulatory effect of HSYA on LTCC through patch-clamp experiments. Bay-K8644 greatly activated calcium channels. HSYA, however, inhibited the higher calcium currents induced by Bay-K8644 (Figure 9). Moreover, the LTCC inhibitor nisoldipine also suppressed the surge in calcium currents. Taken together, the results indicate that HSYA can counteract the activation of calcium channels induced by Bay-K8644.

## 4. Discussion

*Carthamus tinctorius* L. has been used for thousands of years, and its traditional applications include promoting blood circulation, clearing blood stasis, and relieving pain [31, 32]. HSYA, one of the main active ingredients of *Carthamus tinctorius* L., has a flavonoid structure and excellent antioxidant activity [16, 33]. In addition, our team has previously demonstrated that HSYA has a protective effect in rats against MI/RI by regulating the NLRP3 inflammasome and autophagy [12, 13]. However, the currently available evidence is not sufficient to promote HSYA application in the clinical stage. This study further analyzed the specific mechanisms underlying HSYA-mediated cardioprotection from the perspective of calcium channel regulation, aiming to advance the knowledge of HSYA as a treatment against MI/IR.

Myocardial enzyme levels are important for measuring the degree of myocardial cell damage [2]. This study demonstrated that HSYA can significantly attenuate HR-induced AST, CK-MB, and LDH levels in a dose-dependent manner. Apoptosis, a characteristic change occurring in MI/RI, is induced through several mechanisms, including the death receptor pathway, mitochondrial apoptosis pathway, and endoplasmic reticulum stress pathway [34–36]. Here, TUNEL and JC-1 staining revealed that the protective effect of HSYA against MI/RI is related to the inhibition of apoptosis. JC-1 staining reflects the mitochondrial membrane potential, which also suggests that HSYA has a regulatory effect on mitochondrial function [37]. Cardiomyocytes are responsible for the main cardiac task of excitation-contraction coupling [38]. Consistent with previous results, the cardio-ECR detection system used to evaluate the function of hiPSC-CMs showed that HSYA alleviated HR-induced myocardial contraction amplitude, beat rate, field potential amplitude, and duration disorders. Thus, HSYA can alleviate MI/RI damage both to the structure and function of the heart.

In myocardial cells, Ca^2+^ is mainly stored in the sarcoplasmic reticulum and mitochondria, and its concentration in the cytoplasm at rest state is very low [39]. Ca^2+^ can activate a variety of biological enzymes as the second messenger and plays a pivotal role in cardiac excitation-contraction coupling [40]. Here, myocardial ischemia and hypoxia caused a significant increase in the intracytoplasmic [Ca^2+^]_i_ (calcium overload) after cardiomyocyte damage, which was confirmed by calcium fluorescence staining. HSYA however inhibited calcium overload after MI/RI, and reduced MI/RI induced the higher expression of LTCC subunits. LTCC is the main calcium channel type on cardiomyocytes, which is opened when cardiomyocytes are excited [38]. Ca^2+^ then flows into the cell to stimulate the sarcoplasmic reticulum to further release a large amount of Ca^2+^, which constitutes the main calcium flow required for myocardial contraction [41]. Calcium channel blockers are used clinically to protect the heart by blocking this process and inhibiting calcium overload [42]. Our patch-clamp experiments showed that HSYA significantly inhibited the increased calcium current caused by MI/RI, with an effect comparable to that of the LTCC inhibitor nisoldipine. Bay-K8644 is an agonist of LTCC, which can cause hiPSC-CM contraction and field potential dysfunction. Preincubation with HSYA gradually restored the contractile function and intracellular electrical signals of hiPSC-CMs. Patch-clamp experiments also demonstrated that HSYA could inhibit the increase in calcium current caused by Bay-K8644. Collectively, these results indicate that HSYA may directly inhibit LTCC, which could be one mechanism by which HSYA protects against MI/RI and HR injury. In this study, HSYA was administered before MI/RI to reduce myocardial damage. Our research is not limited to MIRI treatment, and preadministration also means that the candidate is potential to have a preventive effect for coronary heart disease. For example, such as the marketed compound Danshen tablets, compound Danshen dripping pills, and Tongxinluo capsules can prevent and treat coronary heart disease and be helpful for patients at risk of cardiovascular disease [43–45]. In fact, in the field of basic research, preadministration of the myocardial ischemia model is a common and recognized method [46, 47].

Because the pathogenesis of MIRI is very complicated, whether HSYA exerts myocardial protection by regulating other mechanisms needs further experimental investigation. Besides, studies have shown that the combination of traditional Chinese medicine and modern western medicine can prevent or improve the occurrence of cardiovascular diseases [48]. Therefore, more in-depth research on the ingredients of Chinese medicine will help clarify the protective mechanism of Chinese medicine on the heart and then develop effective drugs that really solve the pain of cardiovascular disease patients [6, 48]. However, there are some limitations in this study. We used hiPSC-CMs and NPCMs to study the myocardial protection of HSYA in this study. But, compared with human cardiomyocytes, hiPSC-CMs and NPCMs have some differences in membrane potential and calcium current [49, 50]. This study has proved that HSYA has a positive regulatory effect on the LTCC of hiPSC-CMs and NPCMs after MI/RI, so further clinical data are needed for verification.

## 5. Conclusions

In the study, HSYA alleviated MI/RI in rats and HR injury in hiPSC-CMs and NPCMs by inhibiting LTCC, thereby reducing calcium overload. These conclusions fully explained the main mechanism by which HSYA protected myocardial injury caused by MI/RI and provided the basis for preclinical research as an anti-MI/RI drug candidate.

## Data Availability

The data and drug samples used to support the findings of this study are available from the corresponding author upon request.
